# Residual flexibility in the topologically constrained multivalent complex between the GKAP scaffold and LC8 hub proteins

**DOI:** 10.1111/febs.70219

**Published:** 2025-08-22

**Authors:** Eszter Nagy‐Kanta, Zsófia E. Kálmán, Helena Tossavainen, Tünde Juhász, Fanni Farkas, József Hegedüs, Melinda Keresztes, Tamás Beke‐Somfai, Zoltán Gáspári, Perttu Permi, Bálint Péterfia

**Affiliations:** ^1^ Faculty of Information Technology and Bionics Pázmány Péter Catholic University Budapest Hungary; ^2^ Department of Biological and Environmental Science University of Jyvaskyla Finland; ^3^ HUN‐REN Research Centre for Natural Sciences Budapest Hungary; ^4^ Department of Chemistry University of Jyväskylä Finland; ^5^ Institute of Biotechnology, Helsinki Institute of Life Science University of Helsinki Finland

**Keywords:** intrinsically disordered protein, molecular dynamics, postsynaptic density, protein NMR, protein–protein interaction

## Abstract

Guanylate kinase‐associated protein (GKAP) is a large postsynaptic scaffold protein bearing two closely spaced noncanonical binding sites for the bivalent dynein light chain LC8 hub protein. This might allow the formation of heterogeneous complexes with different sizes and topologies. Here, we show that a well‐defined hexameric complex is formed, composed of two GKAP molecules and two LC8 dimers. Using nuclear magnetic resonance (NMR) spectroscopy, we demonstrate that the LC8‐binding segment of GKAP is intrinsically disordered and the flexibility of the linker region is largely retained even in the complex form. Molecular dynamics calculations suggest that, besides the tightly bound residues, the hexamer also exhibits several dynamically interchanging interactions, and that the two LC8 dimers might interact with each other. The flanking regions of the two binding sites on GKAP exhibit different interaction patterns, hinting at additional contacts that might explain the fixed stoichiometry of the assembly. Our results demonstrate that constrained stoichiometry can coexist with substantial flexibility in a multivalent system.

## Graphical abstract

The scaffold protein GKAP and the hub protein LC8 form a well‐defined hexameric complex, with the stoichiometry of 2 GKAP : 2 LC8 dimers. NMR and molecular dynamics calculations confirm that the LC8‐binding segment of GKAP is intrinsically disordered even in the complex form. Flanking regions of the two binding sites on GKAP exhibit different interaction patterns. LC8 dimers might interact with each other.

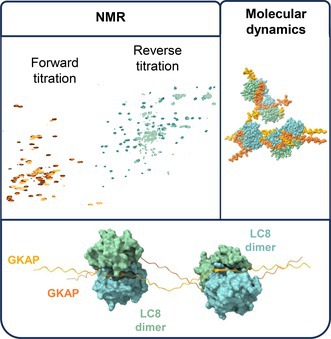

**Abbreviations**
AOP: angular order parameter
BLI: biolayer interferometry
BMRB: Biological Magnetic Resonance Bank
CheSPI: chemical shift secondary structure population inference
DLC1, DLC2: dynein light chain 1, 2
DLS: dynamic light scattering
DSSP, DSSPcont: dictionary of secondary structure in proteins, continuous secondary structure assignment
ECD: electronic circular dichroism
GKAP: guanylate kinase‐associated protein
HSQC: heteronuclear single quantum coherence (spectrum)
IDP/IDR: intrinsically disordered protein/region
ITC: isothermal titration calorimetry
MD: molecular dynamics
*M*_r_
: molar mass
NMDA (NMDAr): *N*‐methyl‐d‐aspartate (receptor)
NMR: nuclear magnetic resonance (spectroscopy)
PDB: Protein Data Bank
POTENCI: prediction of temperature, neighbor and pH corrected shifts for intrinsically disordered proteins
PSD: postsynaptic density
SDS/PAGE: sodium dodecyl sulfate/polyacrylamide gel electrophoresis
SEC: size exclusion chromatography
SLiM: short linear motif
TCEP: tris(2‐carboxyethyl)phosphine hydrochloride

## Introduction

An increasing number of examples suggests that many intrinsically disordered proteins or regions (IDP/IDRs) are capable of multivalent binding and that the simultaneous formation of multiple interactions may be a common feature of IDPs [1]. There are many forms of multivalent assemblies, but the commonality is that a well folded domain participates besides an IDP or a disordered region [2]. Intrinsic disorder is a fundamental feature in the multivalent interactions formed by hub proteins. The ability to bind to a wide range of partner molecules makes hub proteins key components in maintaining the cell homeostasis [3]. The dynein light chain protein LC8 has been identified as a dominant, well‐folded binding partner that can form multivalent complexes with many IDP/IDRs [2]. The two mammalian paralogues DLC1 (or DYNLL1) and DLC2 (or DYNLL2), both aliased as LC8, differ only in six out of 89 residues, and they are fully conserved as orthologues [4, 5]. LC8 was originally recognised as a member of the multi‐subunit dynein motor complex, but it has also been described as interacting with many partners in diverse cellular localisations. LC8 is considered a hub protein involved in many different cellular processes interacting with an extremely high variety of partners (more than 100 verified partners; see *LC8 hub* created by E. J. Barbar *et al*. [6, 7, 8], Table S1). Many LC8 partners are primarily disordered [2] which makes LC8 a prime example of a multivalent folded protein where multispecificity is possible because of its preference for disordered partners [9].

LC8 complexes are found in many different cellular assemblies [2], but the core structural basis for the complex formation is common [10]. Strong LC8 homodimerisation (*K*_d_ ~ 60 nm) [4, 11, 12, 13] is necessary for the functionality, because both sub‐units participate in the formation of the binding pocket. Disordered partners bind to the LC8 dimer through Short Linear Motifs (SLiMs). Motif preferences of LC8‐binding partners have been described in the literature [14, 15, 16, 17]. Two loose consensus sequence classes have been proposed for LC8 partners: [K/R]_−3_X_−2_T_−1_Q_0_T_1_ and G_−2_I_−1_Q_0_V_1_D_2_ with the most conserved glutamine (Q_0_) set as the zero reference point. All known LC8 partners adopt a β‐strand structure upon binding to the LC8 homodimer interface [18]. The LC8 interface contains a shared β‐sheet with five strands (β1, β4, β5, β2 from one subunit, β3 from the other subunit), and a sixth strand is added by β‐sheet augmentation from the binding partner [8, 19], while two α‐helices stabilise this arrangement on the outer surface (α1 and α2). Additionally, the LC8 dimer is symmetric, therefore two identical binding sites are capable of interacting with two ligands, on the opposite sides of the dimer. Details of such interactions, involving both DLC1 and DLC2, are extensively described in the literature, almost exclusively based on LC8 complexes with small peptides. The majority of these contain one LC8 dimer; only a few higher‐order systems have been studied so far (Table S1). All structures of such multivalent complexes, where the LC8‐binding partner contains multiple binding sites, have been solved by X‐ray crystallography. In these cases, crystal contacts between different assemblies are unavoidably present, forming potential stabilising interactions and likely constraining the observed complex geometries. The available NMR studies on several multivalent LC8 partners indicate a high degree of flexibility between consecutive binding sites [20, 21, 22] that is largely retained in the investigated complexes. Remarkably, these complexes are formed ‘in‐register’, meaning that LC8 dimers bind the same binding sites on both instances of the disordered partner molecule, leading to a dominant complex form with well‐defined stoichiometry [3, 21, 22]. In the case of ASCIZ protein, ‘in‐register’ binding is believed to be governed by the different binding affinities of the LC8‐binding sites. For this protein, LC8 binding to a weak, noncanonical site is triggered by the interaction at a nearby canonical site separated by a linker of only three residues, and, based on NMR relaxation experiments, the two closely spaced LC8 dimers likely behave as a single structural unit [21]. These studies primarily focus on partners with canonical binding sites (TQT and [I/V]QT in one case). To our knowledge, in terms of flexibility, no bivalent construct with noncanonical motifs has been characterised yet. In addition, for longer linker regions, the possibility of interactions between consecutive LC8 dimers has not been explored so far.

**Fig. 1:**
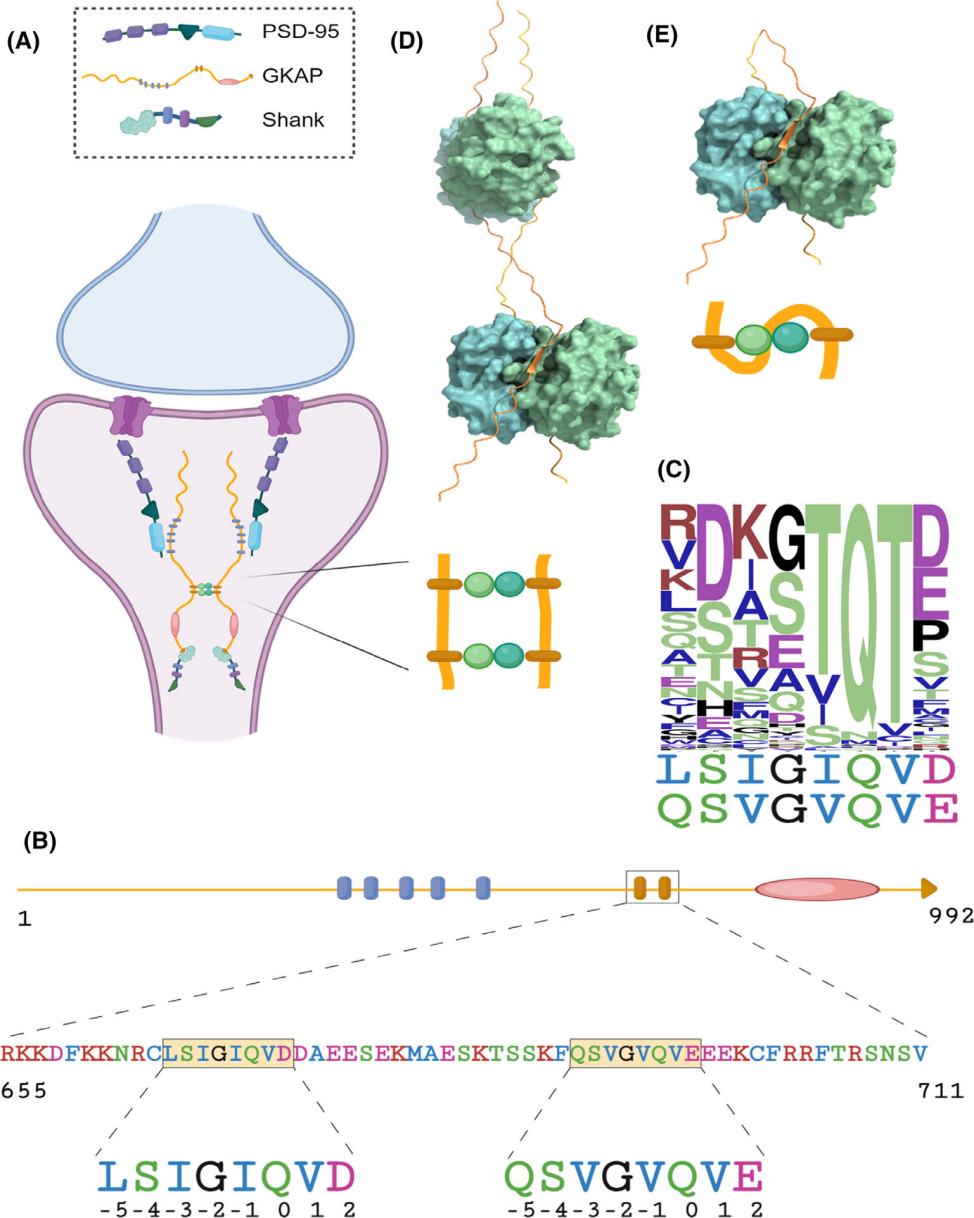
Interaction between GKAP and LC8. (A) GKAP is an abundant postsynaptic scaffold protein involved in the localisation and organisation of NMDA receptor complex. A plausible role of the interaction between GKAP and LC8 is to promote dimerisation; (B) GKAP is predicted to be almost fully disordered with the exception of its C‐terminal GH1 domain. Among others, GKAP contains five binding motifs for PSD‐95 and two LC8‐binding sites. The enlarged segment shows the construct used in this study with the two core LC8‐binding motifs highlighted. The numbers refer to the positions within the binding motif, with relative numbering where Q_0_ is the anchor residue [15]; (C) Sequence logo of 117 LC8‐binding motifs based on LC8 hub [7] and literature review (Table S1), with noncanonical binding motifs of GKAP displayed below the logo. Hydrophobic residues are coloured in blue, polar residues in green, acidic residues in magenta, basic residues in red, all other residues in black. Sequence logo was created with weblogo (https://weblogo.berkeley.edu/ [69]); (D, E) Proposed model of LC8 : GKAP interactions from Moutin et al. with 2 : 2 or 1 : 1 stoichiometry (i.e. 2 GKAP : 2 LC8 dimers or 1 GKAP : 1 LC8 dimer). Panels A, B, D and E were created with Microsoft Office PowerPoint; D and E was based on a structure visualisation made with ucsf chimerax [68].

One of the many known LC8‐partner molecules is GKAP (guanylate kinase‐associated protein, SAPAP1, Dlgap1). It is a postsynaptic protein interacting with several partners, including some of the most important proteins of the postsynaptic density (PSD) like PSD‐95 and the Shank family [23, 24] (Fig. 1A). GKAP is a scaffold protein contributing to the regulation, modulation and enhancement/amplification of signal transduction through the postsynaptic cell via NMDA receptors. GKAP is predicted to be largely disordered throughout its entire length, with high structural flexibility even in the binding regions, making it a perfect candidate to be an adjustable linker molecule in PSD [25]. GKAP harbours two LC8‐binding sites (Fig. 1B) and is likely to be able to bind multivalently to many other partners. The presence of multiple binding sites on GKAP have been experimentally confirmed for the proteins PSD‐95 [26], Arc N‐lobe [27] and Grb2 [28]. The available experimental structures for GKAP are limited to short peptides and its GH1 domain. This domain has been successfully crystallised and its structure consists of four α helices; its function is however unknown [29].

The suspected role of the GKAP‐LC8 interaction is in the organisation of the NMDA receptor complex [30]. This interaction is suggested to be involved in the motor protein‐mediated trafficking, targeting and organisation of the PSD‐95 complex [30, 31]. GKAP as a scaffold molecule is a member of the core complex (PSD‐95/GKAP/Shank complex) organising glutamate receptors and the assembly of PSD [26, 30]. GKAP physically links NMDA receptors and surrounding scaffold molecules to the motor proteins of the cytoskeleton [29, 30].

The existence of the GKAP : LC8 interaction has been described and confirmed multiple times [14, 31, 32, 33]. The binding sites were specified with various methods, first with a yeast two‐hybrid system [31], then later with the pepscan technique [14, 33, 34] and co‐immunoprecipitation [31, 32, 35]. The interaction has been experimentally investigated in vivo within a cellular environment using fluorescence fluctuation microscopy (two‐photon scanning number and brightness, sN&B) [35] and live‐cell confocal‐imaging [36]. Whereas DLC2 and DLC1 are highly related proteins having similar biochemical activities, based on yeast two‐hybrid assays, GKAP seems to prefer DLC2 over DLC1 [31].

GKAP contains two linear motifs, having atypical amino acid composition, which are involved in LC8‐binding. Both binding segments lack the consensus ‘TQT’ anchor region [7], and have atypical residues also in the other positions of the binding region (Fig. 1B,C). The sequence logo shown in Fig. 1C illustrates the frequent occurrence of the TQT motif in typical binding segments along with the diversity of the other positions in the motif. Nevertheless, the conserved binding mode of LC8 to its ligands (via ꞵ‐sheet augmentation) suggests that its interaction with GKAP will also conform to this pattern. GKAP is capable of binding two LC8 dimers, and LC8 dimers are also able to bind to two ligands, therefore there is a possibility of forming hetero‐oligomeric molecular scaffolds. In line with this consideration, Moutin et al. proposed two distinct types of organisation for the GKAP‐LC8 complex. They confirmed the complex formation in vivo [35], but the exact stoichiometry and the atomic structure of the complex is yet to be verified. Their hypothesis is that the complex is most likely formed with a 2 : 2 stoichiometry (i.e. two GKAP monomers and two LC8 dimers, Fig. 1D,E).

In this study we describe the multivalent interaction between the LC8 dimer and the dynein‐binding region of GKAP as characterised by a combination of methods, including NMR spectroscopy. Our analysis provides a comprehensive description on the multivalent GKAP‐LC8‐interaction, including NMR titration measurements in two, complementary setups: ^15^N, ^13^C labeled GKAP titrated with unlabeled LC8 and ^15^N, ^13^C labeled LC8 titrated with unlabeled GKAP. Our results suggest that predominantly hexameric complexes are formed (i.e. two GKAP chains binding two LC8 dimers), which, however, retain some of the flexibility characteristics of free GKAP, allowing the formation of several dynamically interchanging interactions.

## Results

### The LC8‐binding segment of GKAP is disordered

For this study we selected the segment spanning residues 655–711 in *Rattus norvegicus* GKAP (UniProt: P97836‐1) (Fig. 1B). This region is 94.5% identical to the corresponding one in the human GKAP sequence (UniProt: O14490), with one substitution in the flanking, and seven substitutions in the linker region between the binding motifs. This segment, which we refer to as rGKAP_655–711_, contains both LC8‐binding motifs with flanking regions of 10 residues on the N‐terminus and 14 residues on the C‐terminus.

**Fig. 2:**
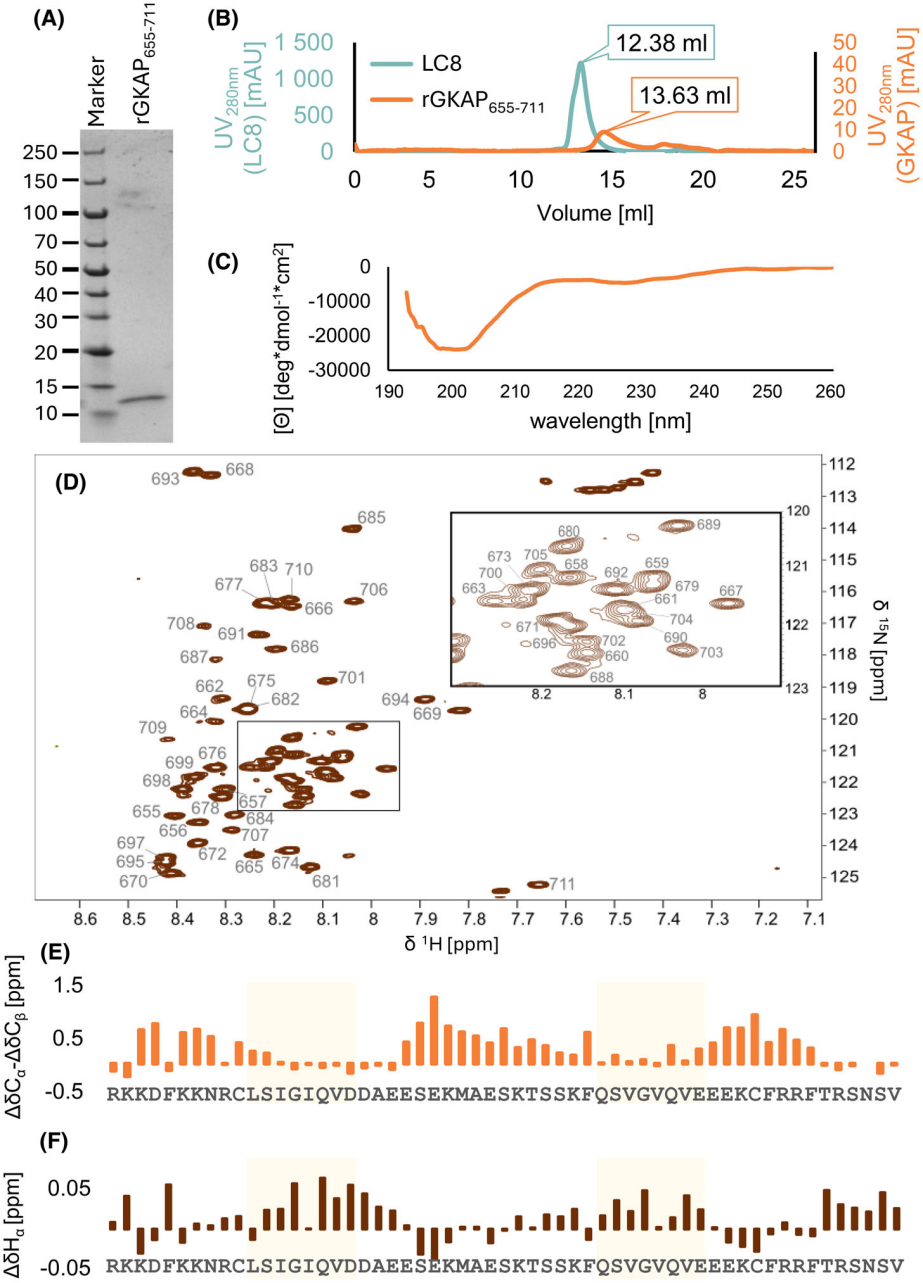
The LC8‐binding segment of GKAP is disordered based on experimental data. All figures show representative results of the experiments. (A) On SDS‐PAGE, GKAP migrates with an apparent molecular mass of 13 kDa instead of 7 kDa, as characteristic of disordered proteins. The gel image presented here is a representative image of seven experiments; (B) Analytical size exclusion chromatogram of the rGKAP_655–711_ and the LC8 dimer for reference, showing larger hydrodynamic dimensions than in the case of an ordered protein. The plot shown is a representative result of two experiments; (C) Far‐UV ECD (electronic circular dichroism) spectrum of rGKAP_655–711_ indicates the absence of a well‐folded structure. The spectrum shown here is a representative result of three experiments; (D) ^1^H‐^15^N HSQC (Heteronuclear Single Quantum Coherence) spectrum of rGKAP_655–711_ exhibits low signal dispersion, typical for disordered regions. Chemical shift assignments are denoted with the corresponding residue number. Inset shows expansion of the highlighted region between 120–123 p.p.m. and 7.9–8.3 p.p.m. for the ^15^N and ^1^H dimensions, respectively. The spectrum presented is a representative result of three independent, altogether six experiments; (E, F) sequential neighbourhood‐, temperature‐ and pH‐corrected secondary chemical shift values shown as a difference for C^α^‐C^β^ (in E) and H^α^ (in F) atoms indicate a disordered structure with slight preferences for extended structure in the binding regions and helical structures outside these. Light yellow colour highlights the two LC8‐binding motifs (LSIGIQVD and QSVGVQVE).

The rGKAP_655–711_ construct exhibits typical properties of an extended IDP, as it namely shows large hydrodynamic dimensions, and it is characterised by low content of ordered secondary structure. The molar mass (*M*_r_) calculated from the encoded amino acid sequence is 7 kDa (7014.88 Da), while on SDS/polyacrylamide gel electrophoresis (SDS/PAGE), the protein migrates more slowly with an apparent *M*_r_ of approximately 13 kDa (Fig. 2A). The observed behaviour of rGKAP_655–711_ in analytical size exclusion chromatography (SEC) also confirmed that its hydrodynamic dimensions are larger than expected for a folded protein. Based on the observed retention volume, the ratio of the measured vs. the theoretical molecular mass was 1.56, which corresponds to a completely unfolded state (Fig. 2B) [37]. The far‐UV electronic circular dichroism (ECD) spectrum of the construct was consistent with this (Fig. 2C).

Complete backbone and partial sidechain NMR chemical shift assignment of rGKAP_655–711_ (100% of the N, H^N^, C′, C^α^, C^β^, H^α^ and H^β^ atoms, and 52% and 61% of the C^γ^ and H^γ^ atoms, respectively) was deposited in BMRB: 52712. The C^β^ chemical shifts of both cysteines (C664 and C701) were 27.9 p.p.m., consistent with the presence of free thiol groups in the buffer containing reducing agent Tris(2‐carboxyethyl)phosphine hydrochloride (TCEP).

Signal dispersion in the ^1^H, ^15^N Heteronuclear Single Quantum Coherence (HSQC) spectrum of the rGKAP_655–711_ construct is ~ 1.2 p.p.m. in the ^1^H and ~ 13 p.p.m. in the ^15^N dimensions, respectively (Fig. 2D). Sequential neighbourhood‐, temperature‐ and pH‐corrected secondary chemical shift values [38] for C^α^‐C^β^ atoms are between −0.2 and +1.2 p.p.m. (Fig. 2E). Secondary chemical shift values for the H^α^ atoms is between −0.2 and +0.6 p.p.m. (Fig. 2F). These observations confirm that the construct is intrinsically disordered along its full length. The calculated differences between C^α^‐C^β^ secondary chemical shifts indicate a slight preference for extended conformation in the first LC8‐binding motif (Fig. 2E, residues 667–674) and varying helical propensity outside the LC8‐binding motifs (residues 656–665, 675–688 and 696–704). CheSPI [39] analysis also shows a mainly disordered structure with no strong preference for stable secondary structure formation, based on the C′, C^α^, C^β^, N and H^N^ chemical shifts (data not shown).

### GKAP binds LC8 at both binding sites simultaneously

**Fig. 3:**
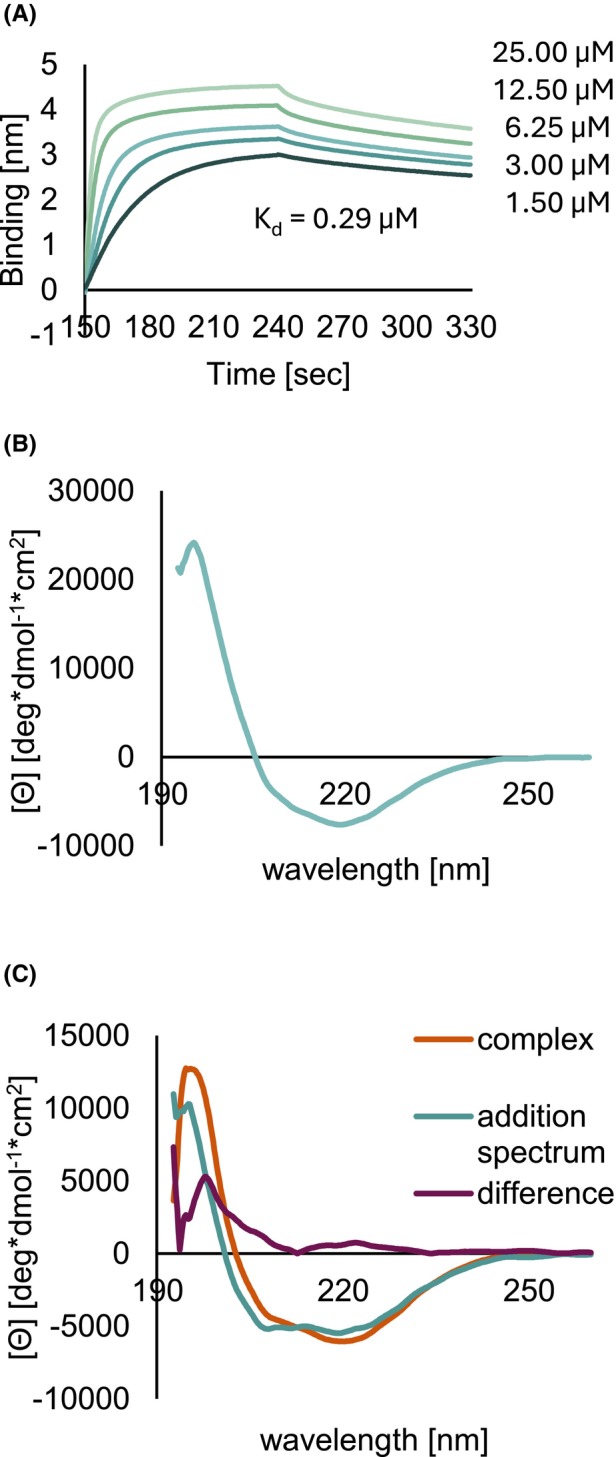
The GKAP : LC8 interaction was investigated with different experimental techniques, describing the binding kinetics, and also the secondary structure propensities of the participating molecules. (A) BLI (Biolayer Interferometry) sensogram for the interaction of rGKAP_655–711_ and LC8, measurement of immobilised GKAP with free LC8. Dissociation constant (*K*_d_) of the interaction proved to be 0.29 μm. The plot presented here is a representative result of three independent experiments; (B, C) Circular dichroism (CD) measurements of the LC8 dimer and the GKAP‐LC8 complex. The plots presented here are representative spectra resulting from two experiments. (B) The CD spectrum of LC8 indicates a well‐folded structure and matches previously reported spectra [41]. (C) The CD spectrum of the GKAP : LC8 complex is slightly different from the sum of the spectra of the individual partners, consistent with the presence of the interaction and the preservation of the folded LC8 structure. The spectrum is not indicative of any large‐scale structural rearrangements.

Biolayer interferometry (BLI) experiments show that LC8‐binding to GKAP follows pseudo‐first‐order kinetics with a single transition (Fig. 3A). The dissociation constant *K*_d_ of the complex is 0.29 μm (*R* ^2^ = 0.9779, aspecific binding: Data not shown). This implies a relatively strong interaction compared to the nNOS peptide binding to LC8 dimer (5.41 ± 0.15 μm measured with isothermal titration calorimetry, ITC), another protein with a noncanonical LC8‐binding motif (having IQV instead of TQT residues in the central region [40], Table S1). Association rate constant (*k*_a_) is 8800 ± 83 m ^−1^·s^−1^, while dissociation rate constant (*K*_d_) is 2.5 × 10^−3^ ± 4.3 × 10^−5^ s^−1^.

**Fig. 4:**
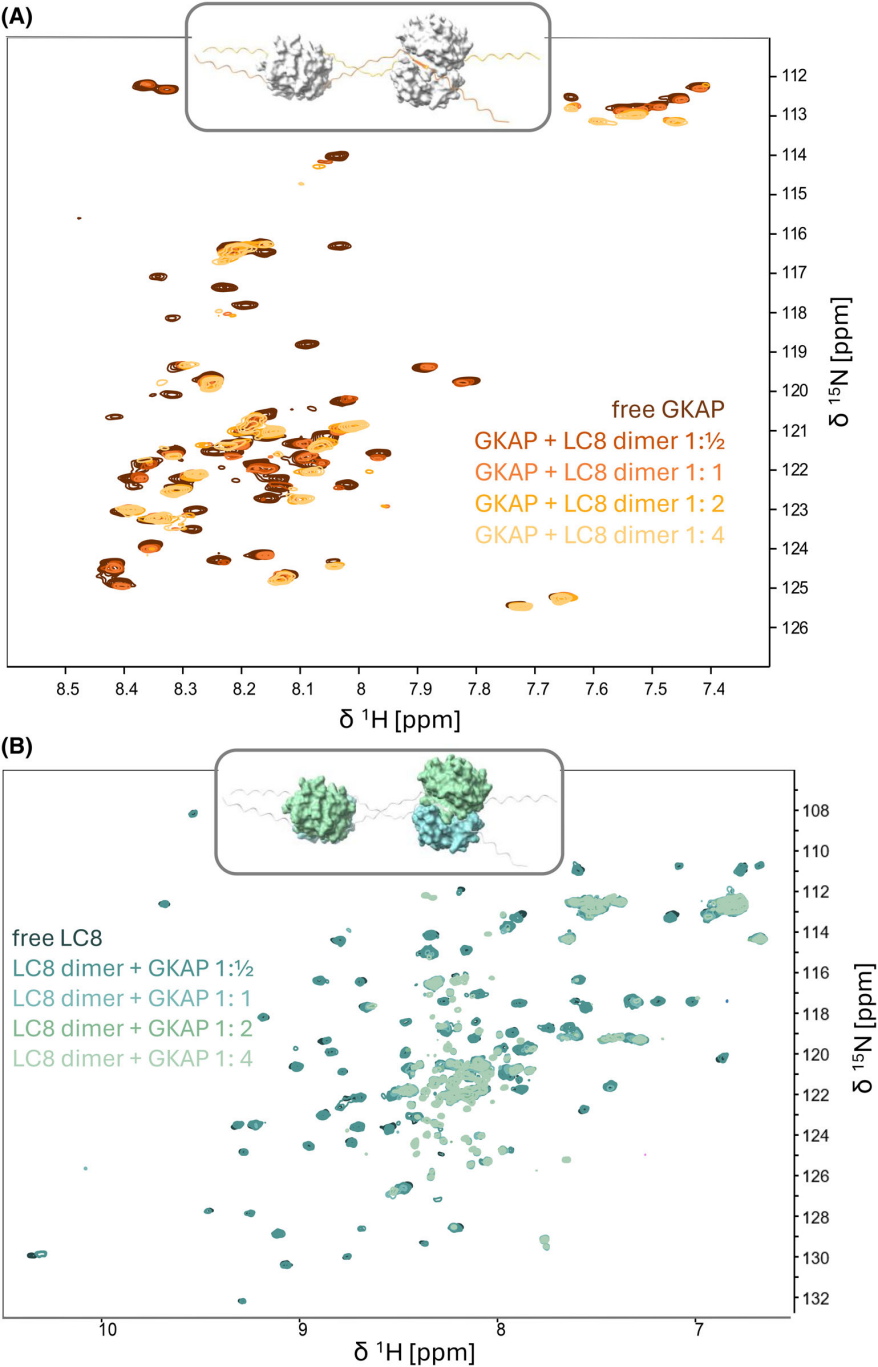
Nuclear magnetic resonance (NMR) titration experiments monitoring the GKAP : LC8 interaction. (A) ‘Forward’ titration: ^1^H, ^15^N, heteronuclear single quantum coherence (HSQC) of GKAP in the presence of LC8 dimers at ratios of 1 : ½, 1 : 1, 1 : 2, 1 : 4. (B) ‘Reverse’ titration: ^1^H, ^15^N HSQC of LC8 dimers at GKAP ratios of 1 : ½, 1 : 1, 1 : 2, 1 : 4; insets show a structural model of the hexameric complex with the ^15^N‐labeled components coloured according to the titration setup.

NMR titration of ^15^N, ^13^C‐labeled rGKAP_655–711_ with unlabeled LC8 (referred to as ‘forward’ titration henceforth to distinguish this setup from the ‘reverse’ titration where the labeling was the opposite, see below, Fig. 4A,B) revealed the involvement of both LC8‐binding sites on GKAP. We monitored binding using ^1^H, ^15^N HSQC at GKAP : LC8 ratios of 1 : ½, 1 : 1, 1 : 2, 1 : 4 (considering the dimeric concentration of LC8). There are residues at both binding sites with clearly disappearing amide N–H peaks starting at an estimated molar ratio of 1 : 1 (equal to two GKAP sites with two LC8 dimers) during titration, with no sign of heterogeneity at the population level. In other words, LC8 seems to bind equally tightly to the two GKAP sites. The last two titration points (GKAP : LC8 dimer 1 : 2 and 1 : 4) are virtually indistinguishable indicating that saturation was reached (Fig. 4A). The limited number of peaks and the position of peaks implies sample homogeneity in the titration endpoints. The observed behaviour of the peaks can be due to the formation of a high molecular weight species or otherwise extensive line broadening due to exchange phenomena occurring at the μs–ms timescale. In either case the simplest interpretation is that the corresponding residues participate in interactions that are not present in the absence of LC8. We note that very similar behaviour is observed in the ‘reverse’ titration for the amide peaks of ^15^N, ^13^C‐labeled LC8 when complexed with unlabeled rGKAP_655–711_, strongly suggesting the formation of a molecular species largely invisible for NMR (Fig. 4B). Results from dynamic light scattering (DLS) experiments are consistent with a largely homogeneous fraction of the GKAP : LC8 complexes formed. The estimated hydrodynamic diameter is in the range of ~ 5 nm, larger than the estimates obtained for the unbound rGKAP_655–711_ segment and the unliganded LC8 dimers (~1–4 and ~ 3–4 nm, respectively). This approximated diameter is also consistent with the calculated molecular weight of ~ 56.6 kDa of a hexamer containing four LC8 chains (two dimers) and two rGKAP_655–711_ segments. Furthermore, Far‐UV ECD spectrum of the GKAP : LC8 complex is slightly different from the sum of the spectra of the individual partners, consistent with the presence of the interaction but not indicative of any large‐scale structural rearrangement in the partners (Fig. 3B).

These observations suggest that the emerging complex has a well‐defined, hexameric stoichiometry with two GKAP segments bound by two LC8 dimers. The complex also presumably has a preferred topology in terms of the arrangement of the binding sites, meaning that one LC8 dimer binds to identical sites on the two GKAP segments, as proposed in Fig. 3C by Moutin et al. [35]. Structural modeling reveals that the 17‐residue linker between the two LC8‐binding sites enables the simultaneous binding of two LC8 dimers along each GKAP segment, arranged in a parallel geometry. Dimensions of our hexameric structural models fall in the range of the experimentally determined size of the complex (data not shown). Our results rule out the possibility of the 1 : 1 stoichiometry (or 2 : 1 according to Moutin et al. [35], Fig. 1D,E), where the two binding motifs of one GKAP molecule interact with one LC8 dimer, and the GKAP disordered linker between the binding motifs is wrapped around the compact dynein dimer.

### The central region of GKAP remains disordered in the complexed state

**Fig. 5:**
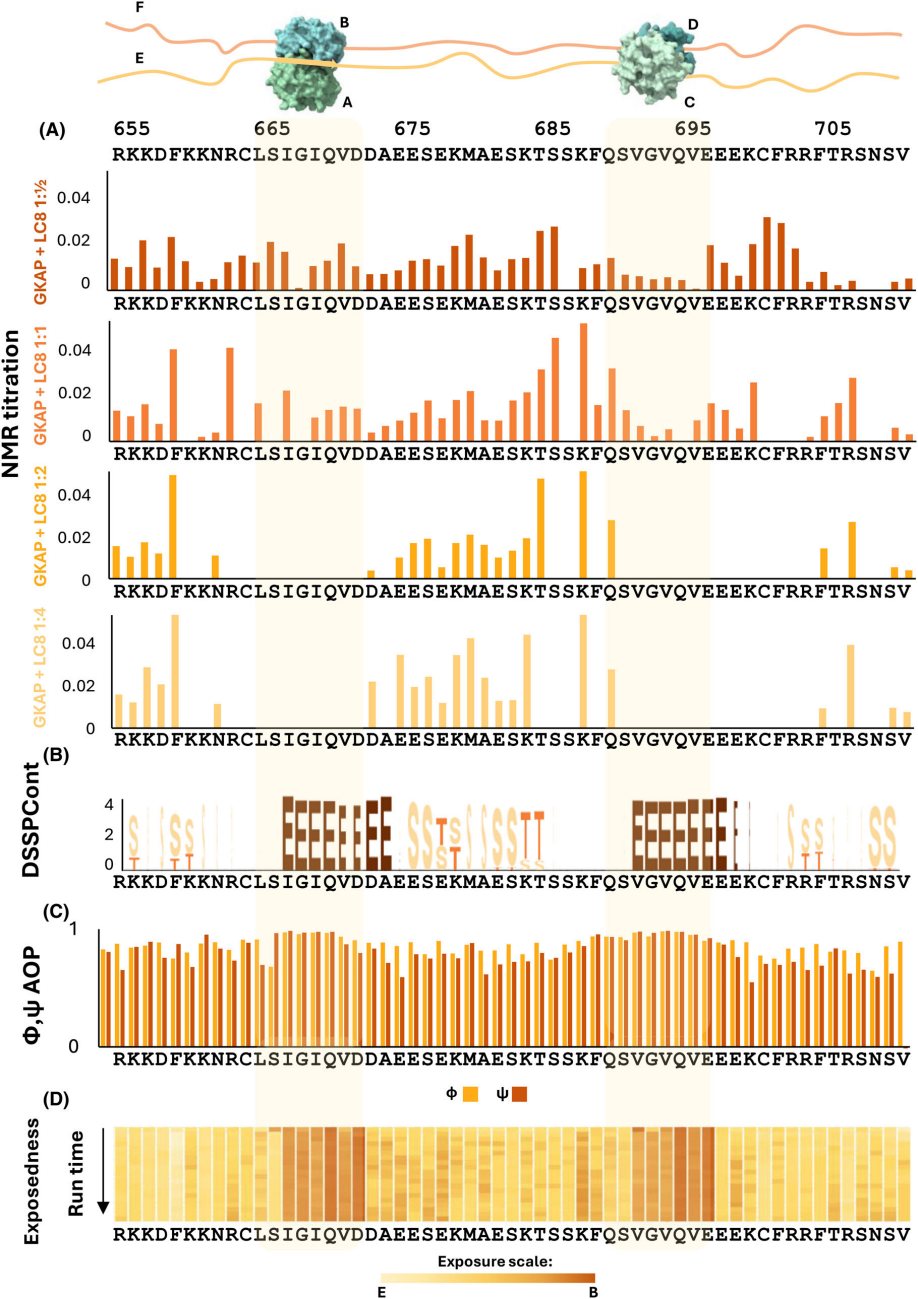
Numerical evaluation of the structural properties of LC8‐bound GKAP. Light yellow colour highlights the two LC8‐binding motifs (LSIGIQVD and QSVGVQVE) throughout the whole figure. The structural assembly (manually edited in Microsoft Office PowerPoint based on a figure created with ucsf chimerax [68]) is shown on the top for illustration purposes; LC8 dimers are not proportional to the length of the manually drawn extended GKAP polypeptide chains. (A) Chemical shift perturbation data of the ‘forward’ titration (migration of the peaks on the spectum) is visualised on the plot for the following stoichiometric settings: GKAP monomers versus LC8 dimers 1 : ½, 1 : 1, 1 : 2, 1 : 4; (B) Representative DSSPCont (Dictionary of Secondary Structure in Proteins, continuous secondary structure assignment) example showing secondary structure elements from one GKAP chain in a selected molecular dynamics run of the complex (E stands for extended, S stands for loop and T stands for turn; for all other chains: data not shown); (C) Angular order parameters (AOP) of the backbone φ and ψ angles calculated for the one GKAP chain in a selected MD (molecular dynamics) run of the complex; (D) Changes in solvent exposure during the simulations of the complex as calculated by DSSP (average of the MD runs for the manually built models).

Curiously, a number of peaks remain virtually unchanged in the ^1^H‐^15^N HSQC spectra of ^15^N‐labeled rGKAP_655–711_ complexed with unlabeled LC8. The majority of these peaks can be mapped to residues in the region between the two LC8‐binding sites, indicating that this part of the polypeptide largely retains its disordered nature even in the complex form (see N and H^N^ chemical shift perturbation data on Fig. 5A). Consequently, the linker region between the two binding sites on rGKAP_655–711_ retains much of its flexibility, and its chemical environment is not considerably different in the bound and the free form.

All amide peaks disappear from their original positions in the neighbourhood of the first binding motif, from K_−10_ to A_4_, with the exception of N_−8_ and D_3_ (see the relative amino acid‐numbering in Fig. 1B). All the peaks disappear in the neighbourhood of the second binding motif, from T_−10_ to N_14_, with the exception of K_−7_, Q_−5_, F_7_, and R_9_. Although intensive signal overlap and strong ambiguity can be observed in the ^1^H, ^13^C HSQC titration spectra (data not shown), the persistence of disappearance of several peaks can be confirmed even when considering their C^α^‐H^α^, and C^β^‐H^β^ peaks. The following residues undoubtedly disappear from both ^1^H‐^15^N HSQC and ^1^H‐^13^C HSQC spectra, with both the C^α^‐H^α^, and C^β^‐H^β^ peaks: C_−6_, L_−5_, I_−3_, G_−2_, I_−1_, Q_0_, D_2_ in the first, and F_−6_, G_−2_, V_−1_, Q_0_, V_1_, E_4_, C_6_, R_9_, T_11_ in the second binding motif. Besides this, several peaks on the N‐terminus and in the linker region between the binding motifs remain visible in the same position with approximately the same intensity during the titration steps as in the free form (Fig. 5A).

Based on molecular dynamics (MD) simulations, in the bound state the two LC8‐binding sites form a β‐strand structure, but they behave slightly differently, especially when also considering their flanking regions. Dictionary of Secondary Structure in Proteins (DSSP) [41] cannot consistently assign regular secondary structural elements to a significant fraction of amino acid residues outside the binding region (in some cases it assigns helices). With DSSPcont (continuous secondary structure assignment, [42]) the main characteristic of the linker seems to be flexible/disordered; a slight preference for helical conformation is however observable in some of our simulations, which is in line with experimental results (Fig. 5B).

**Fig. 6:**
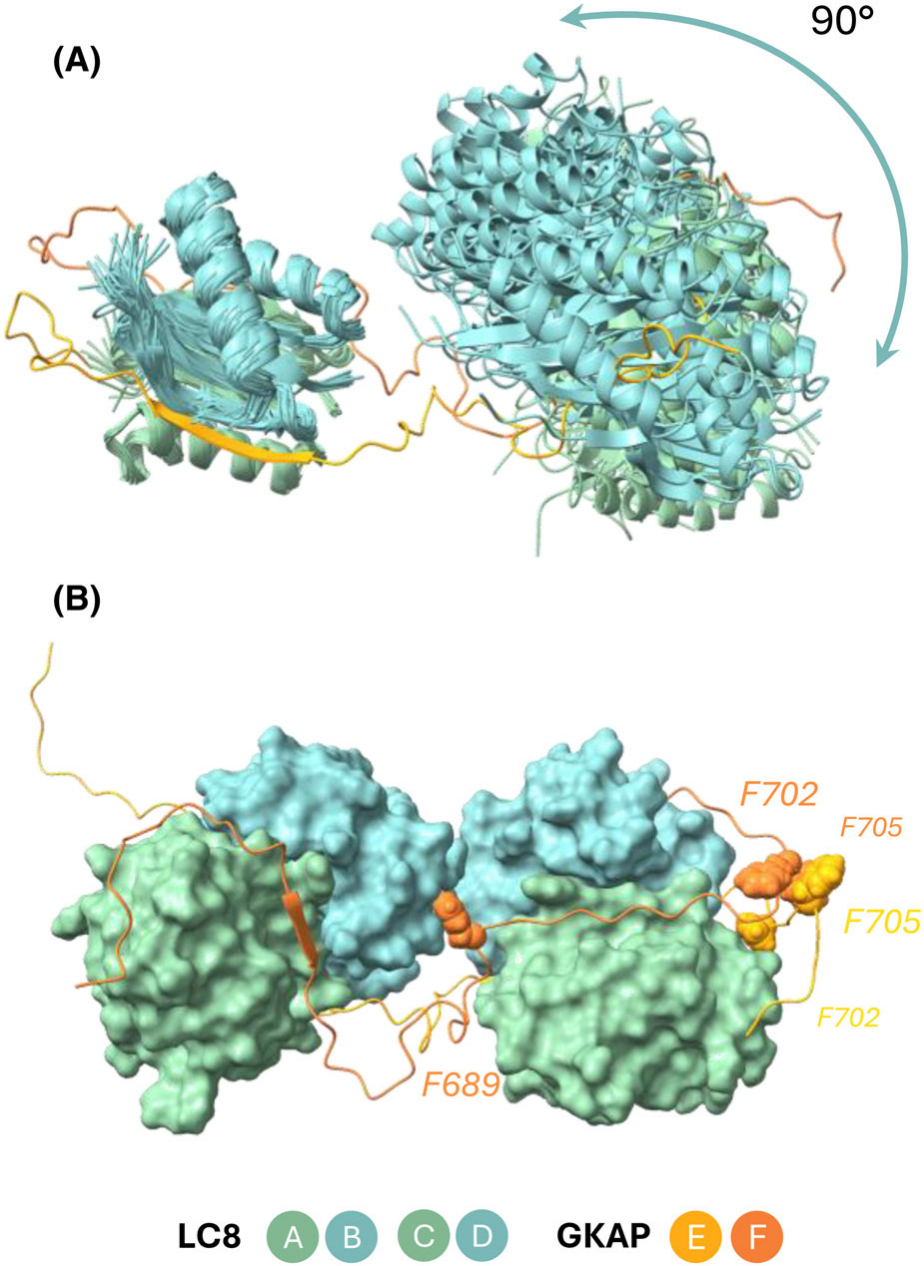
Molecular dynamics results highlight the disordered nature of GKAP (even in complex with LC8). Structural illustrations were created with ucsf chimerax [68]. Chain A–D code LC8 monomer subunits, coloured in green and blue, while chain E and F code GKAP monomer subunits are coloured in yellow and orange. (A) High flexibility of GKAP is illustrated on the figure, the possibility of LC8 dimer – LC8 dimer contact is shown; (B) Phenylalanine residues (F689, F702 and F705) are highlighted with sphere representation, and their spatial position raises the possibility of GKAP‐GKAP contact.

Our MD simulations along with AlphaFold Multimer‐based modeling suggest that the relative orientation of the two LC8 dimers in the hexameric assembly can be highly variable. It is consistent with the presence of a flexible linker between the binding sites (Fig. 6A). However, in our simulations, the typical scenario shows the two LC8 dimers approaching each other and forming contacts, with minimal changes in their relative orientation throughout the remainder of the simulations. In this respect, both these and the various models obtained with AlphaFold Multimer represent largely static snapshots, rather than offering direct insight into the dynamic rearrangement that is plausibly the scenario most consistent with our experimental observations. The retained structural flexibility is also confirmed by the angular order parameters calculated for the backbone φ/ψ angles of GKAP conformers in the MD trajectories. The values are consistently lower outside of the binding regions, indicating higher structural variability (Fig. 5C).

### The two GKAP‐binding sites show a slight difference in LC8‐binding

NMR data show that both GKAP‐binding sites are affected during binding, however, there are differences between their structural properties. For both binding sites the affected peaks reveal the involvement of residues outside the immediate binding site, up to position −10 towards the N‐terminus and at least to position 4 towards the C‐terminus. The flanking regions around the binding sites participate in the structural stabilisation of the interaction, but in different ways. The peaks corresponding to the flanking regions surrounding the binding motifs mainly disappear (with the exception of 2–4 residues) from the ^1^H, ^15^N HSQC titration spectra (Fig. 5A), but with slightly different patterns for the two sites. Two explanations are possible for this phenomenon (a) either these regions are wrapped around the LC8 dimer, losing their flexibility, or (b) the incorporated amino acids participate in intermediate exchange time‐scale events.

The two binding sites show slightly different flexibility as also deducted from DSSPCont, but this is only pronounced in some of the individual runs (Table S2). Some positions in the core motif are more confidently identified as beta‐sheets and others can adopt multiple states. However, these differences are only subtle when considering the average of all runs as shown in Fig. 5B. In the MD simulations, two lysine residues in positions −10 and −9 of the N‐terminal binding region (K660 and K661) seem to be able to form interactions with LC8, but these are not stable, and, most importantly, it is apparently not possible for both residues to be bound simultaneously. The most likely explanation for this is that no backbone conformation is compatible with both side chains being buried. Our hypothesis is that these interactions are dynamically interchanging at the μs–ms time scale, causing line broadening and thus rendering the peaks ‘invisible’ for NMR. No characteristic difference is observed between the core IQV‐and VQV‐binding motifs either in the NMR spectra or in the MD simulations: all corresponding peaks disappear at a 1 : 4 concentration (along with other neighbouring residues) and they also exhibit similar residue‐residue contacts in the trajectories. The common feature is that positions −1 and +1 are completely buried and make contact with LC8 residues forming strands β3 and β4 (Fig. 5D).

Positions −8, −7 and −5 exhibit different behaviour in the two binding sites, despite belonging, in some cases, to chemically similar amino acids. For example, in position −7, the amide peak of R663 in the first binding site completely disappears, whereas the peak of the corresponding K688 in the second site shows a moderate position shift. The simulations show that these positions are buried over time in the first binding site and maintain significant interactions with LC8. The N‐terminal flanking region of the first binding motif participates loosely in the interaction. F689 in position −6 with regard to the second binding site can either contact the LC8 dimer at the second site or that which binds to the first, N‐terminal site. The observed flexibility of the linker region suggests that this residue might not be tightly locked in a single position, rather undergoing binding and unbinding events (Fig. 6B). Even in the binding motif, a possible fast exchange phenomenon can be observed in the −4 position serine, but due to lack of bound‐form assignments this cannot be unambiguously concluded.

The differences are even more pronounced for the C‐terminal flanking segments. Residues in positions 5–14 are affected only in the second, C‐terminal‐binding site, most of them disappearing completely (Fig. 5A). The corresponding positions relative to the first N‐terminal‐binding site coincide with the linker between the sites and are only moderately affected, indicative of segments remaining largely disordered as described above. These differences reveal the importance of investigating constructs with longer flanking regions in contrast with studies focusing only on the minimal binding regions. Our MD calculations suggest that this region could potentially be buried and come into significant proximity to LC8, but no consistent pattern is observed, in line with the experimentally observed dynamic nature of the region. The most remarkable difference between the two binding sites is that the C‐terminal‐binding site and its C‐terminally located flanking region seem to be involved in complex formation via possible exosite contacts. In the case of the N‐terminal‐binding motif, residues immediately after the minimal binding site, starting from position +3 – with the sole exception of A_4_–, give rise to N‐H peaks in the HSQC spectrum of the complex, indicating residual flexibility, while residues after the C‐terminal site become undetectable by NMR. This kind of difference cannot be readily explained by our MD simulations, where both flanking segments appear partially buried (Fig. 5D). However, it has been noted previously that LC8 bears a positively charged surface patch where it interacts with these positions [20, 43], and thus the different arrangement of negatively charged residues, especially the series of three glutamates at positions 2–4 after the C‐terminal site, might contribute to the observed differences.

In several simulated conformers, the two GKAP segments make extensive hydrophobic contacts with each other using two phenylalanine side chains, F702 and F705, in the C‐terminal flanking region after the second binding site. This suggests that the two GKAP chains might form specific interactions with each other, facilitated by the binding of the LC8 proteins (Fig. 6B). It might be speculated that this interaction might serve as a trigger for the formation and stabilisation of an otherwise weak and noncanonical coiled coil segment.

### The two LC8 dimers interact with each other in the hexameric assembly

We have performed a partial Backbone Assignment of the LC8 dimer. Although multiple data sets for LC8 chemical shifts are available in BMRB, none of the available assignments were performed in a TCEP‐containing buffer. ‘Reverse’ titration, using ^15^N, ^13^C‐labeled LC8 and unlabeled rGKAP_655–711_ revealed profound changes in the ^1^H, ^15^N HSQC spectrum of LC8. At the monomer LC8 : GKAP ratios of 1 : ½ (equaling GKAP : LC8 dimer ratios of 2 : 2, where hypothetically all LC8 dimers are in bound form) and at titration points with higher excess of GKAP, most LC8 peaks disappear. However, many peaks in the middle ^1^H region of the spectrum are still present. We investigated the system further by recording sensitive, overnight ^1^H, ^15^N spectra, in which peaks belonging to both the ^15^N‐labeled LC8 along with those of the unlabeled GKAP were observed. These latter peaks are located in positions matching the remaining peaks in the ‘forward’ titration, i.e. the ^1^H, ^15^N HSQC spectrum of ^15^N‐labeled GKAP in complex with unlabeled LC8. Moreover, ^15^N‐filtered ^1^H spectra clearly indicate that these peaks arise from ^1^H, ^14^N groups, i.e. unlabeled GKAP, in the ‘reverse’ titration experiments. This confirms the disappearance of the majority of LC8 ^1^H, ^15^N peaks (Fig. 4B).

The remaining LC8 peaks include those corresponding to the amide NH of residues D3, R4, A6 and S88, which can be unambiguously assigned. A few more peaks also clearly belong to the LC8 dimer; however, based on the acquired spectra they could not be reliably assigned (due to unfeasibility of 3D experiments of the complex). Our MD calculations indicate that there are some residues, mainly in helices α1 and α2, that are not involved in either dimerisation‐ or ligand‐binding. On the other hand, MD simulations suggest that a multitude of interactions might be formed between the LC8 dimers binding to different binding sites. These can involve different residues at the N‐terminus, helices α1 and α2, the strand β1 and also loop regions. Thus, it is likely that the behaviour of some the amide peaks belonging to residues outside the ligand‐binding site can be explained by intermolecular interactions between the two LC8 dimers. On the other hand, unaffected peaks again indicate residual flexibility also in this partner. Residues R4 (near the N‐terminus) and N51 in different LC8 dimers are brought into physical contact in one of our MD trajectories. This might be due to the fact that the N‐terminal region of the LC8 chains remains slightly flexible, also confirmed by the NMR ‘reverse’ titration experiments.

The general observation is that peptide‐binding does not significantly change the conformation of LC8 apo‐form [10]. Our MD simulation of LC8 indicates that the structure of LC8 dimers is not significantly affected by ligand‐binding.

## Discussion

Our NMR and ECD data clearly demonstrate the intrinsically disordered nature of the LC8‐binding segment of GKAP. The observed high flexibility of rGKAP_655–711_ indicates it to be perfectly suitable for organising the NMDA receptor complex in the postsynaptic cells. The flexibility of GKAP, especially in the LC8‐bound state, makes it possible to establish physical connection between PSD proteins located in versatile mutual orientations, keeping the protein network dynamic but still interconnected.

The LC8 : GKAP interaction involves two kinds of bivalent partners, the globular LC8 dimers and the highly flexible GKAP segments. The behaviour of the NMR peaks in both the ‘forward’ and ‘reverse’ titrations along with DLS and SEC‐based size estimates points to the emergence of a single species with a large molecular weight that is largely ‘invisible’ to NMR investigation. In contrast, specific regions both on GKAP and LC8 retain considerable flexibility and still give rise to observable peaks in NMR. The different behaviour of peaks together with MD simulations hint at the presence of potential dynamic rearrangements within the complex. MD simulations are expected to yield insights into the behaviour of the system. In our case, because of the scarcity of NMR data on the complex, no quantitative agreement can be enforced between experiment and simulation. It cannot be necessarily expected that a conventional simulation recapitulates all structural and dynamical aspects of the complex, thus, we have chosen to explore the MD trajectories to see whether any of them can provide clues for selected observations. Some general phenomena are consistently reproduced by the simulations (e.g. retained flexibility between binding sites, see Fig. 6A); however, many unique events and interactions can be observed only occasionally, at different points of the separate runs.

The disappearance of the peaks both in GKAP in and close to the binding sites and in LC8, where the majority of the residues are affected, can be due to the formation of a large molecular weight species, emergence of a dynamic process leading to intermediate exchange at the NMR time scale, or both. Our DLS measurements, along with additional data, suggest that the stoichiometry of the complex is hexameric, encompassing almost 500 residues with a molecular mass of 56 kDa. This points to the large size of the assembly as a primary factor explaining the invisibility of the peaks. However, our structural models suggest that the two LC8 dimers might be able to interact with each other at various surfaces, therefore, a dynamic interchange between different binding modes can not be completely excluded. Such an interchange could also contribute to the disappearance of the peaks. It should also be noted that among the few peaks remaining visible from the LC8 molecules, some appear at different positions relative to the free state, clearly indicating changes in the chemical environment of the corresponding residues. Considering all our observations, including CD and NMR spectroscopy and molecular modeling, the most plausible explanation is that there are additional intermolecular interactions present in the hexamer outside the primary LC8 : GKAP‐binding sites. This is somewhat reminiscent of the behaviour of two neighbouring LC8 dimers at the QT2‐ and QT3‐binding sites on the protein ASCIZ, although there the two binding sites are separated by a much shorter linker region [21]. In that case, the two LC8 dimers were suggested to form a species acting as a single unit in terms of molecular tumbling. The scenario with GKAP is somewhat different because of the longer linker that remains partially disordered even in the complexed state, possibly allowing multiple kinds of interactions between the neighbouring LC8 dimers.

The significance of the disappearing peaks can be evaluated in the light of the previously published literature on the structures of LC8 and its ligands. Interestingly, the first (N‐terminal) binding region of GKAP has a L in position −5. According to Rapali et al. [16], in natural binding partners this position seems not to favour any specific residue type. In contrast, in their in vitro evolutional study there is a preference for apolar (mainly V, but also M/I/L) or long aliphatic (R/K) amino acids and this results in increased affinity. In the second (C‐terminal) binding region, position −5 is occupied by Q which does not belong to any of the aforementioned categories. Serine in position −4 is a shared feature between the two binding motifs, and besides backbone hydrogen bond‐forming, it might also participate in sidechain hydrogen‐bonding based on our results and literature data [16, 20, 44].

Based on previous studies, the residues from positions −8 to +4 in the LC8‐binding motif are relevant for the binding affinity in a monovalent interaction with LC8 [14, 45]. In the case of short peptides representing the minimal binding motif, the presence or absence of a residue in the −5 position can change the dissociation constant by ten‐ to hundred‐fold [16]. Our results emphasise the relevance of the regions flanking the binding motif (from −10 to +4, and even to +14 in the case of the second binding motif), refining the results from previous efforts to characterise the minimal sequence segments required for binding (LSIGIQVD, QSVGVQVE, from −5 to +2). Our most intriguing finding is that while the linker between the two sites retains its flexibility, a remarkably long C‐terminal flanking region of the second binding motif is involved in the interaction.

Like GKAP, the nNOS peptide is an IQV motif partner. By performing a mutational analysis on nNOS (−6 MKDTGIQVDRDL +5) it was found that mutating the two hydrophobic amino acids flanking the central glutamine residue to asparagines (i.e. Ile in −1 and Val in +1 position) completely abolishes the interaction. They found that I57, F62, F73, A82, L84 and F86 of LC8 interact with these two amino acids [10], which is consistent with our MD results. On the ‘reverse’ NMR titration spectra I57, L84 and F86 disappear from the spectra (F62 and F73 cannot be assigned).

The C‐terminal flanking region of the second binding motif participates in the complex formation. Negatively charged residues at the +2 and +3 positions are able to form electrostatic interactions with spatially close charged amino acids of LC8 [20, 43]. The PDB: 1F96 structure is the only available NMR structure for a ligand bound LC8 dimer. In this case, a longer C‐terminal region was part of the construct. Based on the deposited NMR distance restraints, Gln in +3 and Gln in +5 are in contact with residues in the β1–β2 loop and the strands β3 and β4 of LC8 [8]. Our observations are in line with these results and it is thus feasible that residues following the core‐binding motif also form interactions with LC8 but this might largely depend on the actual amino acid sequence.

One of the main features of the LC8 hub protein is that it forms multivalent interactions by binding to multiple motifs, which in most cases involves two consecutive binding sites on their partner molecule. In general, it seems that the canonical ‘TQT’ motif containing partners bind LC8 with the highest affinity. As the sequence diverges from this pattern, binding affinity decreases (resulting in higher *K*_d_). As multivalent interactions are usually formed to strengthen the generally weak binding mediated by SLiMs, it is plausible to speculate that the presence of consecutive non‐TQT motifs result in higher overall affinity compared to that of a single motif, possibly due to avidity. *K*_d_ values (Table S1) of the LC8 interactors clearly indicate an increase in binding strength in favour of partners with two binding motifs over single binding motifs, by at least one order of magnitude. Our results (*K*_d_ = 0.29 μm) are in line with this trend. In general, monovalent ‘TQT’ binding partners have *K*_d_ values between the range of 0.1 and 27.9 μm, while in the case of dimer partners, *K*_d_ can be as low as 0.003 or 0.007 μm. The multivalent Chica protein containing four binding motifs has a *K*_d_ value of 0.4 μm, similar to GKAP measured here. For the GKAP‐LC8 hexameric complex, the observed *K*_d_ is smaller than for the similar ‘IQV’ nNOS peptide (sequence: DTGIQVD, *K*_d_ = 5.4 μm) and the association and dissociation rate constants also show a higher binding affinity (for nNOS peptide, *k*_a_ = 58 400 m ^−1^·s^−1^ and *K*_d_ = 4.37 × 10^−1^ s^−1^) [40]. More details and binding affinity values of LC8‐binding events are listed in Table S1. In the vast majority of experimentally proven multivalent binding LC8 partners, the two binding regions are < 50 amino acids apart. The GKAP : LC8 interaction also follows this pattern. The LC8 hub database [7] lists 14 bivalent or multivalent LC8‐interacting proteins. LC8 was proposed to act as a dimerisation engine [46], and some of its interaction partners, like Swallow, were validated [47] and others were predicted to undergo oligomerisation via coiled‐coils. Examples include Ana2, Bassoon, Kibra and Nup159 [17, 48, 49, 50]. For Ana2, the coiled‐coil region is located between the two binding sites [17].

Our systematic search for multivalent protein : LC8 interactions resulted in five similar structures in the PDB, with a longer protein segment (27–33 residues) incorporating two distinct LC8‐binding motifs in complex with two LC8 molecules. Two of them (PDB: 3FM7 [51] and PDB: 2PG1 [52]) are complexes of one DLC1 dimer, one DLC Tctex‐type dimer and two disordered dynein intermediate chains having two LC8‐binding sites. The PDB: 3GLW structure is a quasi‐hexamer generated via translational and rotational transformations of a DLC1 monomer and an intermediate chain partner peptide unit [51]. The fourth structure contains DLC1 dimers with the protein Panoramix (PDB: 7K3J [53]) and the fifth is a DLC1‐SAO‐1 complex (PDB: 7Y8W [54]) (Table S1). All these PDB structures were solved by X‐ray crystallography and all these complexes have an elongated shape with a parallel topology, meaning each LC8 dimer either binds two N‐terminal or two C‐terminal sites on the partners. This arrangement is consistent with our model based on NMR and other data. In the X‐ray structures, the linker regions between the LC8‐binding sites adopt an extended conformation, and the LC8 dimers binding to the same partners typically do not interact with each other (crystal contacts between different hexamers are possible). However, these features might be the results of crystal packing, and the flexibility of these segments cannot necessarily be directly assessed based on these structures.

Here we provide experimental evidence for GKAP that the linker region between the two binding motifs remains highly flexible even when both binding sites are occupied and the heterohexamer is formed (Fig. 6A). In the PDB structures mentioned above, 3–9 residues are located between the two binding motifs, while in GKAP the linker region consists of 17 residues. For Dynactine and ASCIZ a flexible region between binding sites was analysed with NMR [21, 22]. In addition, none of the LC8 partner proteins in the solved structures have amino acid compositions similar to that of GKAP in their binding motifs (harbouring the canonical TQT or TQV anchor sequence in the binding motif, except for SAO‐1, having VAT and CQT).

Analysis of the linker regions between LC8‐binding motifs was performed by the group of E. J. Barbar, using the multivalent IDP ASCIZ having seven LC8‐binding motifs [3, 21]. They conclude that shorter linker regions cause increased rigidity of the resulting complexes. Based on their proposal, the longer linker region resulting in higher flexibility might explain the functionality of the complex as an assembly‐scaffold, a regulator‐modulator of the postsynaptic density signal transduction pathway initiated at NMDA receptors [21]. They also proposed a classification of linker lengths as a short, a long, and a ‘mid‐length’ in bivalent/multivalent LC8 partners [3]. According to this, GKAP is a mid‐length bivalent ligand, and here we propose that this results in the formation of a single well‐defined complex with LC8.

The Barbar group also investigated the Chica‐LC8 interaction, where four binding motifs are present on the Chica protein. The Chica protein was proved to be intrinsically disordered by NMR, but solution‐state studies of its complexes with LC8 were hampered by solubility issues. Structures of LC8 with peptides corresponding to individual binding motifs have been solved by X‐ray crystallography (PDB: 5E0L, PDB: 5E0M [20]). The LC8‐binding motifs on the Chica protein show clearly distinct sequence patterns, also leading to differences in their binding properties. They found that the *K*_d_ value of the multivalent binding (when four binding motifs are included in the sequence) equals the *K*_d_ value belonging to the peptide‐binding with the strongest affinity, and that the multimer with multiple bound LC8 dimers does not show enhanced stability. It was proposed that the presence of additional binding sites promotes self‐association. In‐register binding for the investigated ASCIZ protein construct was explained on the basis of preferential binding to the site denoted QT2. In this case, interactions between consecutive LC8 dimers were suggested [21], that might be rationalised on the basis of the very short (three residues) linker between consecutive sites.

In the GKAP construct investigated here, chemical shift perturbations do not indicate clear preference of one binding site over the other during the titration experiments. This is consistent with the high similarity between the two noncanonical binding sites on GKAP. Thus, the formation of the stoichiometric, ‘in‐register’ complex is likely driven by other mechanisms. Our molecular dynamics calculations suggest that the two LC8 dimers can interact with each other, and our NMR data are consistent with such a scenario. However, a clear binding preference cannot be established; this interaction might thus be dynamic and the disappearance of the LC8 peaks might indicate intermediate exchange between different transient complexes involving multiple interfaces. Other factors contributing to the formation of such a complex might be the interactions mediated by GKAP residues outside the immediate binding sites, like F689 that might make alternative contacts with both LC8 dimers.

Although there is no coiled coil predicted with high confidence in GKAP, the output of COILS [55] with a 21‐residue window size hints at the presence of a weak or noncanonical short coiled coil between residues 715–735 (towards the C‐terminal end of the second binding motif, data not shown). It might be speculated that LC8‐binding might trigger the formation of a more extended interaction between GKAP monomers, possibly even forming a short coiled coil, consistent with the behaviour of several other LC8 partners and the dimerisation‐promoting role of LC8 [46]. This kind of interaction between the GKAP molecules could also provide an explanation for the preferred stoichiometry and arrangement of the hexameric assembly, as well as the observed binding kinetics.

Our results demonstrate the considerable added value when combining the power of NMR with molecular dynamic calculations to study complex, flexible systems. The unexpected revelations related to the role of the flanking regions in the interaction highlights the importance of investigating protein segments, including all binding motifs and their neighbouring regions instead of short peptides. Examining the residue‐level connections between the postsynaptic density scaffold GKAP and the hub protein LC8 forms the basis of understanding their role and mechanism of action in the postsynaptic density. The flexibility of the hexameric system described here might indicate their functionality as an assembly scaffold or modulator‐regulator complex in the NMDA receptor‐initiated signal transduction pathway in postsynaptic cells.

## Materials and methods

### Protein expression and purification

The GKAP construct was designed to include both LC8‐binding motifs of GKAP with extended flanking regions (10 residues on the N‐terminus, 14 residues on the C‐terminus). The segment‐spanning residues 655–711 in the *R. norvegicus* GKAP isoform 3 ‘GKAP1a’ (UniProt: P97836‐5) was selected. The insert was picked up from a cDNA (ORF template) kindly provided by Enora Moutin. The insert was amplified for cloning and ligated into NdeI and XhoI sites of a pEV plasmid that is an altered pET‐15b vector (Novagen, Madison, WI, USA) that contains an N‐terminal 6xHis tag and a tobacco etch virus (TEV) protease cleavage site. The actual construct contains four extra residues (GSHM) at the N‐terminus after TEV cleavage, remaining from the expression tag.

The plasmid construct containing the *R. norvegicus* DYNLL2 gene (UniProt: Q78P75, 100% identical to the human orthologue, UniProt: Q96FJ2) was kindly provided by L. Nyitray. This cDNA was also cloned in the pEV vector and thus also contains an N‐terminal 6xHis tag, the TEV protease cleavage site and four residues (GSHM) at the N‐terminus remaining from the expression tag.

Both protein constructs were produced in BL21 (DE3) cells (Novagen). Cells were transformed with the vectors described above and grown in LB Broth Low Salt media (Duchefa Biochemie, BH Haarlem, the Netherlands), or in in‐house prepared M9 minimal media containing ^13^C‐labeled d‐glucose (U‐13C6, 99%, CLM‐1396; Cambridge Isotope Laboratories Inc., Andever, MA, USA) and ^15^N‐labeled NH_4_Cl (^15^N, 99%, NLM‐467; Cambridge Isotope Laboratories Inc.) as the only carbon and nitrogen source, respectively. Protein expression was induced with 1 mm of IPTG (Isopropyl β‐D‐1‐thiogalactopyranoside, 26600.06; SERVA, Heidelberg, Germany) at 6 MFU cell density and the recombinant proteins were expressed at 20°C overnight. Centrifuged cell pellets were stored at −20°C until further usage.

Cell pellets were lysed by ultrasonic homogenisation in 10% cell suspension using a lysis buffer (50 mm NaPi, 300 mm NaCl, pH 7.4). In the case of rGKAP_655–711_, denaturing‐renaturing IMAC purification was applied using an NGC 10 Medium‐Pressure Chromatography System (Bio‐Rad Laboratories Inc., Hercules, CA, USA). After homogenisation and centrifugation, the pellet was dissolved in denaturing buffer (6 m GdnHCl, 50 mm sodium phosphate), and loaded on a 5 mL EconoFit Nuvia™ IMAC column (Bio‐Rad Laboratories Inc.), then proteins bound to the column were renatured with native buffer (50 mm sodium phosphate, 20 mm NaCl, pH 7.4). After a washing step with 50 mm, elution was performed with 250 mm imidazole, and was followed by His‐tag removal with TEV protease (in‐house produced and purified). For LC8, the same purification protocol was used but after ultrasonic homogenisation and centrifugation, the supernatant was immediately purified with IMAC Nuvia™ Ni‐affinity column.

Protein samples were concentrated by ultrafiltration using Amicon® Ultra‐15 ultracel‐3K Centrifugal Filters (Merck Millipore Ltd., Tullagreen, Carrigtowhill, Ireland) with 3 kDa molecular weight cut‐off value, and the buffer was changed to a low salt sodium phosphate buffer (50 mm sodium phosphate, 20 mm NaCl, pH 6.0). Proteins were further purified by ion exchange chromatography, using 5 mL High Q column (Bio‐Rad Laboratories Inc.) with the same buffer (50 mm sodium phostphate, 20 mm NaCl, pH 6.0; recombinant proteins were collected in the flow through fraction). After another step of protein concentration, where proteins were further purified with size exclusion chromatography (SEC) on a Superdex™ 75 Increase 10/300 GL column (Cytiva, Uppsala, Sweden), the buffer was changed to 50 mm sodium phosphate, 20 mm NaCl, pH 6.0. Later 0.02% NaN_3_, and 5 mm TCEP (pH 7.4) (Thermo Fisher Scientific Inc., Waltham, MA, USA) was added to every sample individually.

The concentration of LC8 was measured by its absorbance at 280 nm using a NanoDrop2000 photometer, while the concentration of GKAP was measured with Qubit™ Protein Assay Kit (Invitrogen, Life Technologies Corporation, Eugene, OR, USA). The recombinant proteins' purity and exact molecular weight was analysed by SDS/PAGE and LC–MS. The SDS/PAGE gels were evaluated with the software gelanalyzer 23.1.1 (available at www.gelanalyzer.com by Istvan Lazar Jr., PhD and Istvan Lazar Sr., PhD, CSc).

### Analytical size exclusion chromatography (SEC) measurements

In the analytical SEC measurements, the standard proteins: BSA, myoglobin, RNAse A, B12 vitamin were dissolved in the same buffer as GKAP and LC8 (50 mm sodium phosphate, 20 mm NaCl, pH 6.0, 5 mm TCEP) in 200, 2500, 3000 and 300 μm concentration, respectively. Proteins were injected one by one in the same volume (500 μL) as GKAP and LC8 to a Superdex™ 75 Increase 10/300 GL column (Cytiva), and the flowspeed was between 0.8 and 1 mL·min^−1^.

### Far‐UV CD spectroscopy

Proteins were measured by ECD spectroscopy using JASCO J‐1500 spectrometer (JASCO Corporation, Tokyo, Japan). ECD spectra were recorded at 20°C using 0.1 cm path length J/21 quartz cuvette (Hellma absorption cell; Hellma GmbH & Co. KGHellma, Müllheim, Baden Württemberg, Germany) and a 300 μL sample with the following settings: 195–260 nm spectral range, 50 nm·min^−1^ scanning speed, 1 nm bandwidth, 0.2 nm step size, 0.5 s response time and three scans of accumulation and baseline correction. rGKAP_655–711_ was diluted to 6.5 μm in 50 mm sodium phosphate buffer, 20 mm NaCl, pH 6.0 containing 5 mm TCEP. Free LC8 was measured in the very same buffer, diluted to 11.25 μm concentration (considering monomers). The complex was created with the same molar concentrations (i.e. 6.5 μm concentration of GKAP with 11.25 μm concentration of LC8). Thus, we expect that the majority of the molecules is present as part of the complex, which is the dominant species under the conditions used. Comparison of the CD spectra was performed under the assumption that the signal solely represents the complex, and it should be noted that even if this is not completely true, this does not affect our conclusions obtained from the qualitative analysis as described.

### Biolayer interferometry

Biolayer interferometry analysis was carried out on a BLItz™ Protein Detection System device by FortéBio (Menlo Park, CA, USA). Prior to experiments, all Ni‐NTA biosensors (FortéBio) were hydrated in kinetic buffer (50 mm sodium phosphate, 20 mm NaCl, pH 6.0 + 20 mm imidazole, 0.1% BSA, 0.02% Tween 20, 0.02% NaN_3_) at 25°C for 10 min. The ligand, the 6xHis‐tagged rGKAP_655–711_ protein was immobilised on the surface of the biosensor at a 5 μg·mL^−1^ concentration in kinetic buffer for 90 s. The initial baselines were then recorded in kinetic buffer for 30 s. The association and dissociation sensorgrams of LC8 dimers at concentrations ranging from 3.125 to 50 μm were recorded in kinetic buffer for 90 s each. The equilibrium dissociation constant (*K*_d_) was determined from the BLI data using the global fitting method provided in the data analysis software. The curves were adjusted with a background spectrum (with 0 added ligand). To check for aspecific binding, the experiment was repeated with the same settings but without immobilising the 6xHis‐tagged rGKAP_655–711_ construct on the sensor surface in the first step. During the evaluation of the aspecific binding experiment, a baseline was measured with kinetic buffer only.

### Dynamic light scattering

The exact procedure of dynamic light scattering experiments has been published elsewhere [56]. Key points of the measurements are included in this text. Particle size and size distribution of GKAP, LC8 dimer and their complex were measured at 20°C using a W130i dynamic light scattering device (DLS, Avid Nano Ltd., High Wycombe, UK) with a diode laser (660 nm) and a photodiode detector. 80 μL samples were used in low‐volume disposable cuvettes with 1 cm path‐length (UVette; Eppendorf Austria GmbH, Vienna, Austria). The time‐dependent autocorrelation function was measured for 10 s, repeated 10 times. Data analysis was performed using i‐size 3.0 software (Avid Nano Ltd., High Wycombe, UK).

### NMR measurement conditions

The exact procedure of NMR experiments has been published elsewhere [57]. Key points of the protocol are included in this text. NMR experiments were acquired using 0.06–0.5 mm ^15^N, ^13^C‐labeled protein samples in 2/98% D_2_O/H_2_O at pH 6.0. Chemical shifts were referenced to external 2,2‐dimethyl‐2silapentane‐5‐sulfonic acid (DSS). All data were acquired at 298.15 K on a Bruker AVANCE III HD 800 MHz spectrometer (Bruker BioSpin Corporation, Billerica, MA, USA), equipped with a TCI ^1^H/^13^C/^15^N Z‐gradient cryoprobe. Data was collected and processed with topspin version 3.5.7 (Bruker Corporation, Billerica, MA, USA).

The following experiments were used in the resonance assignment of rGKAP_655–711_: ^1^H‐^15^N HSQC, constant‐time ^1^H‐^13^C HSQC‐aliphatic, constant‐time ^1^H‐^13^C HSQC‐aromatic, HNCACB, HN(CO)CACB, HNCO, HBHA(CO)NH, (H)CC(CO)NH, H(CCCO)NH [58, 59] and i(HCA)CO(CA)NH [60]. In the case of LC8, partial backbone assignment of the ^1^H‐^15^N‐HSQC was performed mainly in comparison with BMRB: 15076 [43], with the support of HNCACB and HN(CO)CACB spectra. This was needed for the evaluations of the titration ^1^H‐^15^N HSQC spectra, to identify the disappearing and visible peaks corresponding to the free LC8 peaks. NMR titration experiments were conducted with the unlabeled partner molecules at titration points visible on Fig. 3.

ccpnmr analysis v3.1 (Collaborative Computing Project for NMR, University of Leicester, Department of Molecular and Cell Biology, UK) software was used for the chemical shift assignment.

Chemical shift perturbation values were calculated with the following formula:

$$\Delta ppm\;=\;\sqrt{{\left(\Delta \delta_{HN}\right)}^{2}+{\left(\Delta \delta_{N}\times \alpha \right)}^{2}}$$

where α = 0.14.

### Model building and MD simulations

For the initial model building, PDB: 2XQQ [16] was used as a template. The extended disordered regions were modeled using Dipend and Modeler (Chimera) [61, 62]. Another set of models were generated using AlphaFold in the complex modeling setting in the Colabfold notebook [63]. FoldX was applied before MD simulation.

Computations were performed on the Komondor supercomputer. The models were simulated with gromacs [64] using explicit solvent classical MD. AMBER99SB‐ILDN protein force field was used; the box setting was a minimum distance of 1.0 n. For the initial model, five parallel 200 ns runs were carried out (md_200_1, md_200_2, md_200_3, md_200_4, md_200_5). The AlphaFold generated models each had a 200 ns run time (AF1, AF2, AF3, AF4, AF5). From the ten 200 ns long production run, 10 × 20 000 snapshots were taken.

The MD generated models were analysed as follows: R Secondary structure was predicted using DSSP and DSSPcont consecutively [41, 65]. Exposure was calculated from DSSP. Radius of gyration was calculated with an in‐house script. φ/ψ angles were calculated for each model using phipsi.py script from UCSF and further analysed with an in‐house script. For all other calculations and file‐formatting we used python 3 codes written in our group. Coiled‐coil prediction was made using wagga‐wagga webserver (10.1093/bioinformatics/btu700). The most representative peptide was collected using molmol [66].

The residue level molecular interactions were analysed using voronota [67], from which the average atomic distances were obtained. For visualisation we used chimerax, weblogo and jalview [68, 69, 70].

Any additional information required to repeat the experiments or reanalyse the data reported in this paper is available from the lead contact upon request.

## Conflict of interest

The authors declare no conflict of interest.

## Author contributions

ZG created the concept of the study; ZG, BP, TJ, TB‐S and PP designed the methodology; EN‐K, ZEK, HT, TJ, FF, JH, MK and PP performed the experiments; EN‐K, ZEK, HT, TJ, PP and ZG analysed the data; ZEK and ZG performed molecular dynamics simulations; BP, PP, TB‐S and ZG provided funding and resources; EN‐K, ZEK and ZG wrote the original draft of the manuscript; EN‐K and ZEK created the figures and tables; and BP, PP, HT, TJ, TB‐S and ZG made manuscript revisions.

## Supporting information


**Table S1.** Motif sequence and binding affinity (*K*_d_) of LC8‐binding partners.
**Table S2.** Results of the DSSPcont analysis on the individual molecular dynamics runs.

## Data Availability

The data that support the findings of this study are openly available in BMRB at https://bmrb.io/, reference number 52712.

## References

[1] Sipko EL , Chappell GF & Berlow RB (2024) Multivalency emerges as a common feature of intrinsically disordered protein interactions. Curr Opin Struct Biol 84, 102742. 38096754 10.1016/j.sbi.2023.102742

[2] Clark SA , Jespersen N , Woodward C & Barbar E (2015) Multivalent IDP assemblies: unique properties of LC8‐associated, IDP duplex scaffolds. FEBS Lett 589(19 Pt A), 2543–2551. 26226419 10.1016/j.febslet.2015.07.032 PMC4586992

[3] Walker DR , Jara KA , Rolland AD , Brooks C , Hare W , Swansiger AK , Reardon PN , Prell JS & Barbar EJ (2023) Linker length drives heterogeneity of multivalent complexes of hub protein LC8 and transcription factor ASCIZ. Biomolecules 13, 404. 36979339 10.3390/biom13030404 PMC10046861

[4] Rapali P , Szenes Á , Radnai L , Bakos A , Pál G & Nyitray L (2011) DYNLL/LC8: a light chain subunit of the dynein motor complex and beyond. FEBS J 278, 2980–2996. 21777386 10.1111/j.1742-4658.2011.08254.x

[5] Pfister KK , Shah PR , Hummerich H , Russ A , Cotton J , Annuar AA , King SM & Fisher EMC (2006) Genetic analysis of the cytoplasmic dynein subunit families. PLoS Genet 2, e1. 16440056 10.1371/journal.pgen.0020001 PMC1331979

[6] Erdős G , Szaniszló T , Pajkos M , Hajdu‐Soltész B , Kiss B , Pál G , Nyitray L & Dosztányi Z (2017) Novel linear motif filtering protocol reveals the role of the LC8 dynein light chain in the hippo pathway. PLoS Comput Biol 13, e1005885. 29240760 10.1371/journal.pcbi.1005885 PMC5746249

[7] Jespersen N , Estelle A , Waugh N , Davey NE , Blikstad C , Ammon Y‐C , Akhmanova A , Ivarsson Y , Hendrix DA & Barbar E (2019) Systematic identification of recognition motifs for the hub protein LC8. Life Sci Alliance 2, e201900366. 31266884 10.26508/lsa.201900366 PMC6607443

[8] Hall J , Hall A , Pursifull N & Barbar E (2008) Differences in dynamic structure of LC8 monomer, dimer, and dimer‐peptide complexes. Biochemistry 47, 11940–11952. 18942858 10.1021/bi801093k

[9] Teilum K , Olsen JG & Kragelund BB (2021) On the specificity of protein‐protein interactions in the context of disorder. Biochem J 478, 2035–2050. 34101805 10.1042/BCJ20200828 PMC8203207

[10] Fan J , Zhang Q , Tochio H , Li M & Zhang M (2001) Structural basis of diverse sequence‐dependent target recognition by the 8 kDa dynein light chain. J Mol Biol 306, 97–108. 11178896 10.1006/jmbi.2000.4374

[11] Estelle AB , George A , Barbar EJ & Zuckerman DM (2023) Quantifying cooperative multisite binding in the hub protein LC8 through Bayesian inference. PLoS Comput Biol 19, e1011059. 37083599 10.1371/journal.pcbi.1011059 PMC10155966

[12] Benison G , Chiodo M , Karplus PA & Barbar E (2009) Structural, thermodynamic, and kinetic effects of a phosphomimetic mutation in dynein light chain LC8. Biochemistry 48, 11381–11389. 19863079 10.1021/bi901589w PMC2821902

[13] Lightcap CM , Sun S , Lear JD , Rodeck U , Polenova T & Williams JC (2008) Biochemical and structural characterization of the Pak1‐LC8 interaction. J Biol Chem 283, 27314–27324. 18650427 10.1074/jbc.M800758200 PMC2556000

[14] Rodríguez‐Crespo I , Yélamos B , Roncal F , Albar JP , Ortiz de Montellano PR & Gavilanes F (2001) Identification of novel cellular proteins that bind to the LC8 dynein light chain using a pepscan technique. FEBS Lett 503, 135–141. 11513870 10.1016/s0014-5793(01)02718-1

[15] Benison G , Karplus PA & Barbar E (2008) The interplay of ligand binding and quaternary structure in the diverse interactions of dynein light chain LC8. J Mol Biol 384, 954–966. 18948118 10.1016/j.jmb.2008.09.083 PMC4294432

[16] Rapali P , Radnai L , Süveges D , Harmat V , Tölgyesi F , Wahlgren WY , Katona G , Nyitray L & Pál G (2011) Directed evolution reveals the binding motif preference of the LC8/DYNLL hub protein and predicts large numbers of novel binders in the human proteome. PLoS One 6, e18818. 21533121 10.1371/journal.pone.0018818 PMC3078936

[17] Slevin LK , Romes EM , Dandulakis MG & Slep KC (2014) The mechanism of dynein light chain LC8‐mediated oligomerization of the Ana2 centriole duplication factor. J Biol Chem 289, 20727–20739. 24920673 10.1074/jbc.M114.576041 PMC4110283

[18] Nyarko A , Hall J , Hall A , Hare M , Kremerskothen J & Barbar E (2011) Conformational dynamics promote binding diversity of dynein light chain LC8. Biophys Chem 159, 41–47. 21621319 10.1016/j.bpc.2011.05.001

[19] Remaut H & Waksman G (2006) Protein‐protein interaction through beta‐strand addition. Trends Biochem Sci 31, 436–444. 16828554 10.1016/j.tibs.2006.06.007

[20] Clark S , Nyarko A , Löhr F , Karplus PA & Barbar E (2016) The anchored flexibility model in LC8 motif recognition: insights from the Chica complex. Biochemistry 55, 199–209. 26652654 10.1021/acs.biochem.5b01099 PMC6995037

[21] Reardon PN , Jara KA , Rolland AD , Smith DA , Hoang HTM , Prell JS & Barbar EJ (2020) The dynein light chain 8 (LC8) binds predominantly “in‐register” to a multivalent intrinsically disordered partner. J Biol Chem 295, 4912–4922. 32139510 10.1074/jbc.RA119.011653 PMC7152752

[22] Jie J , Löhr F & Barbar E (2015) Interactions of yeast dynein with dynein light chain and dynactin: general implications for intrinsically disordered duplex scaffolds in multiprotein assemblies. J Biol Chem 290, 23863–23874. 26253171 10.1074/jbc.M115.649715 PMC4583017

[23] Verpelli C , Schmeisser MJ , Sala C & Boeckers TM (2012) Scaffold proteins at the postsynaptic density. Adv Exp Med Biol 970, 29–61. 22351050 10.1007/978-3-7091-0932-8_2

[24] Lowenthal MS , Markey SP & Dosemeci A (2015) Quantitative mass spectrometry measurements reveal stoichiometry of principal postsynaptic density proteins. J Proteome Res 14, 2528–2538. 25874902 10.1021/acs.jproteome.5b00109 PMC5597335

[25] Droogers WJ & MacGillavry HD (2023) Plasticity of postsynaptic nanostructure. Mol Cell Neurosci 124, 103819. 36720293 10.1016/j.mcn.2023.103819

[26] Zhu J , Zhou Q , Shang Y , Li H , Peng M , Ke X , Weng Z , Zhang R , Huang X , Li SSC *et al*. (2017) Synaptic targeting and function of SAPAPs mediated by phosphorylation‐dependent binding to PSD‐95 MAGUKs. Cell Rep 21, 3781–3793. 29281827 10.1016/j.celrep.2017.11.107

[27] Hallin EI , Bramham CR & Kursula P (2021) Structural properties and peptide ligand binding of the capsid homology domains of human arc. Biochem Biophys Rep 26, 100975. 33732907 10.1016/j.bbrep.2021.100975 PMC7941041

[28] Wu C , Ma MH , Brown KR , Geisler M , Li L , Tzeng E , Jia CYH , Jurisica I & Li SS‐C (2007) Systematic identification of SH3 domain‐mediated human protein‐protein interactions by peptide array target screening. Proteomics 7, 1775–1785. 17474147 10.1002/pmic.200601006

[29] Tong J , Yang H , Eom SH , Chun C & Im YJ (2014) Structure of the GH1 domain of guanylate kinase‐associated protein from *Rattus norvegicus* . Biochem Biophys Res Commun 452, 130–135. 25152391 10.1016/j.bbrc.2014.08.073

[30] Moutin E , Raynaud F , Fagni L & Perroy J (2012) GKAP‐DLC2 interaction organizes the postsynaptic scaffold complex to enhance synaptic NMDA receptor activity. J Cell Sci 125(Pt 8), 2030–2040. 22328512 10.1242/jcs.098160

[31] Naisbitt S , Valtschanoff J , Allison DW , Sala C , Kim E , Craig AM , Weinberg RJ & Sheng M (2000) Interaction of the postsynaptic density‐95/guanylate kinase domain‐associated protein complex with a light chain of myosin‐V and dynein. J Neurosci 20, 4524–4534. 10844022 10.1523/JNEUROSCI.20-12-04524.2000 PMC6772433

[32] Haraguchi K , Satoh K , Yanai H , Hamada F , Kawabuchi M & Akiyama T (2000) The hDLG‐associated protein DAP interacts with dynein light chain and neuronal nitric oxide synthase. Genes Cells 5, 905–911. 11122378 10.1046/j.1365-2443.2000.00374.x

[33] Lajoix A‐D , Gross R , Aknin C , Dietz S , Granier C & Laune D (2004) Cellulose membrane supported peptide arrays for deciphering protein‐protein interaction sites: the case of PIN, a protein with multiple natural partners. Mol Divers 8, 281–290. 15384421 10.1023/b:modi.0000036242.01129.27

[34] Navarro‐Lérida I , Martínez Moreno M , Roncal F , Gavilanes F , Albar JP & Rodríguez‐Crespo I (2004) Proteomic identification of brain proteins that interact with dynein light chain LC8. Proteomics 4, 339–346. 14760703 10.1002/pmic.200300528

[35] Moutin E , Compan V , Raynaud F , Clerté C , Bouquier N , Labesse G , Ferguson ML , Fagni L , Royer CA & Perroy J (2014) The stoichiometry of scaffold complexes in living neurons – DLC2 functions as a dimerization engine for GKAP. J Cell Sci 127(Pt 16), 3451–3462. 24938595 10.1242/jcs.145748

[36] Manneville J‐B , Jehanno M & Etienne‐Manneville S (2010) Dlg1 binds GKAP to control dynein association with microtubules, centrosome positioning, and cell polarity. J Cell Biol 191, 585–598. 21041448 10.1083/jcb.201002151 PMC3003329

[37] Uversky VN (2002) What does it mean to be natively unfolded? Eur J Biochem 269, 2–12. 11784292 10.1046/j.0014-2956.2001.02649.x

[38] Nielsen JT & Mulder FAA (2018) POTENCI: prediction of temperature, neighbor and pH‐corrected chemical shifts for intrinsically disordered proteins. J Biomol NMR 70, 141–165. 29399725 10.1007/s10858-018-0166-5

[39] Nielsen JT & Mulder FAA (2021) CheSPI: chemical shift secondary structure population inference. J Biomol NMR 75, 273–291. 34146207 10.1007/s10858-021-00374-w

[40] Radnai L , Rapali P , Hódi Z , Süveges D , Molnár T , Kiss B , Bécsi B , Erdödi F , Buday L , Kardos J *et al*. (2010) Affinity, avidity, and kinetics of target sequence binding to LC8 dynein light chain isoforms. J Biol Chem 285, 38649–38657. 20889982 10.1074/jbc.M110.165894 PMC2992297

[41] Kabsch W & Sander C (1983) Dictionary of protein secondary structure: pattern recognition of hydrogen‐bonded and geometrical features. Biopolymers 22, 2577–2637. 6667333 10.1002/bip.360221211

[42] Andersen CA , Bohr H & Brunak S (2001) Protein secondary structure: category assignment and predictability. FEBS Lett 507, 6–10. 11682049 10.1016/s0014-5793(01)02910-6

[43] Benison G , Karplus PA & Barbar E (2007) Structure and dynamics of LC8 complexes with KXTQT‐motif peptides: swallow and dynein intermediate chain compete for a common site. J Mol Biol 371, 457–468. 17570393 10.1016/j.jmb.2007.05.046

[44] Gallego P , Velazquez‐Campoy A , Regué L , Roig J & Reverter D (2013) Structural analysis of the regulation of the DYNLL/LC8 binding to Nek9 by phosphorylation. J Biol Chem 288, 12283–12294. 23482567 10.1074/jbc.M113.459149 PMC3636912

[45] Bodor A , Radnai L , Hetényi C , Rapali P , Láng A , Kövér KE , Perczel A , Wahlgren WY , Katona G & Nyitray L (2014) DYNLL2 dynein light chain binds to an extended linear motif of myosin 5a tail that has structural plasticity. Biochemistry 53, 7107–7122. 25312846 10.1021/bi500574z

[46] Barbar E (2008) Dynein light chain LC8 is a dimerization hub essential in diverse protein networks. Biochemistry 47, 503–508. 18092820 10.1021/bi701995m

[47] Wang L , Hare M , Hays TS & Barbar E (2004) Dynein light chain LC8 promotes assembly of the coiled‐coil domain of swallow protein. Biochemistry 43, 4611–4620. 15078108 10.1021/bi036328x

[48] Fejtova A , Davydova D , Bischof F , Lazarevic V , Altrock WD , Romorini S , Schöne C , Zuschratter W , Kreutz MR , Garner CC *et al*. (2009) Dynein light chain regulates axonal trafficking and synaptic levels of Bassoon. J Cell Biol 185, 341–355. 19380881 10.1083/jcb.200807155 PMC2700376

[49] Rayala SK , den Hollander P , Manavathi B , Talukder AH , Song C , Peng S , Barnekow A , Kremerskothen J & Kumar R (2006) Essential role of KIBRA in co‐activator function of dynein light chain 1 in mammalian cells. J Biol Chem 281, 19092–19099. 16684779 10.1074/jbc.M600021200

[50] Nyarko A , Song Y , Nováček J , Žídek L & Barbar E (2013) Multiple recognition motifs in nucleoporin Nup159 provide a stable and rigid Nup159‐Dyn2 assembly. J Biol Chem 288, 2614–2622. 23223634 10.1074/jbc.M112.432831 PMC3554928

[51] Hall J , Karplus PA & Barbar E (2009) Multivalency in the assembly of intrinsically disordered dynein intermediate chain. J Biol Chem 284, 33115–33121. 19759397 10.1074/jbc.M109.048587 PMC2785153

[52] Williams JC , Roulhac PL , Roy AG , Vallee RB , Fitzgerald MC & Hendrickson WA (2007) Structural and thermodynamic characterization of a cytoplasmic dynein light chain‐intermediate chain complex. Proc Natl Acad Sci USA 104, 10028–10033. 17551010 10.1073/pnas.0703614104 PMC1885999

[53] Schnabl J , Wang J , Hohmann U , Gehre M , Batki J , Andreev VI , Purkhauser K , Fasching N , Duchek P , Novatchkova M *et al*. (2021) Molecular principles of Piwi‐mediated cotranscriptional silencing through the dimeric SFiNX complex. Genes Dev 35, 392–409. 33574069 10.1101/gad.347989.120 PMC7919418

[54] Zhang D , Yang H , Jiang L , Zhao C , Wang M , Hu B , Yu C , Wei Z & Tse YC (2022) Interaction between DLC‐1 and SAO‐1 facilitates CED‐4 translocation during apoptosis in the *Caenorhabditis elegans* germline. Cell Death Discov 8, 441. 36323675 10.1038/s41420-022-01233-9 PMC9630320

[55] Lupas A , Van Dyke M & Stock J (1991) Predicting coiled coils from protein sequences. Science 252, 1162–1164. 2031185 10.1126/science.252.5009.1162

[56] Singh P , Szigyártó IC , Ricci M , Gaál A , Quemé‐Peña MM , Kitka D , Fülöp L , Turiák L , Drahos L , Varga Z *et al*. (2023) Removal and identification of external protein corona members from RBC‐derived extracellular vesicles by surface manipulating antimicrobial peptides. J Extracell Biol 2, e78. 38938416 10.1002/jex2.78 PMC11080927

[57] Tossavainen H , Salovaara S , Hellman M , Ihalin R & Permi P (2020) Dispersion from cα or NH: 4D experiments for backbone resonance assignment of intrinsically disordered proteins. J Biomol NMR 74, 147–159. 31932991 10.1007/s10858-020-00299-w PMC7080685

[58] Sattler M (1999) Heteronuclear multidimensional NMR experiments for the structure determination of proteins in solution employing pulsed field gradients. Prog Nucl Magn Reson Spectrosc 34, 93–158.

[59] Permi P & Annila A (2004) Coherence transfer in proteins. Prog Nucl Magn Reson Spectrosc 44, 97–137.

[60] Mäntylahti S , Tossavainen H , Hellman M & Permi P (2009) An intraresidual i(HCA)CO(CA)NH experiment for the assignment of main‐chain resonances in ^15^N, ^13^C labeled proteins. J Biomol NMR 45, 301–310. 19768387 10.1007/s10858-009-9373-4

[61] Yang Z , Lasker K , Schneidman‐Duhovny D , Webb B , Huang CC , Pettersen EF , Goddard TD , Meng EC , Sali A & Ferrin TE (2012) UCSF Chimera, MODELLER, and IMP: an integrated modeling system. J Struct Biol 179, 269–278. 21963794 10.1016/j.jsb.2011.09.006 PMC3410985

[62] Harmat Z , Dudola D & Gáspári Z (2021) DIPEND: an open‐source pipeline to generate ensembles of disordered segments using neighbor‐dependent backbone preferences. Biomolecules 11, 1505. 34680137 10.3390/biom11101505 PMC8534045

[63] Mirdita M , Schütze K , Moriwaki Y , Heo L , Ovchinnikov S & Steinegger M (2022) ColabFold: making protein folding accessible to all. Nat Methods 19, 679–682. 35637307 10.1038/s41592-022-01488-1 PMC9184281

[64] Van Der Spoel D , Lindahl E , Hess B , Groenhof G , Mark AE & Berendsen HJC (2005) GROMACS: fast, flexible, and free. J Comput Chem 26, 1701–1718. 16211538 10.1002/jcc.20291

[65] Touw WG , Baakman C , Black J , te Beek TAH , Krieger E , Joosten RP & Vriend G (2015) A series of PDB‐related databanks for everyday needs. Nucleic Acids Res 43, D364–D368. 25352545 10.1093/nar/gku1028 PMC4383885

[66] Koradi R , Billeter M & Wüthrich K (1996) MOLMOL: a program for display and analysis of macromolecular structures. J Mol Graph 14, 51–55, 29–32. 8744573 10.1016/0263-7855(96)00009-4

[67] Olechnovič K & Venclovas C (2014) Voronota: a fast and reliable tool for computing the vertices of the Voronoi diagram of atomic balls. J Comput Chem 35, 672–681. 24523197 10.1002/jcc.23538

[68] Pettersen EF , Goddard TD , Huang CC , Meng EC , Couch GS , Croll TI , Morris JH & Ferrin TE (2021) UCSF ChimeraX: structure visualization for researchers, educators, and developers. Protein Sci 30, 70–82. 32881101 10.1002/pro.3943 PMC7737788

[69] Crooks GE , Hon G , Chandonia J‐M & Brenner SE (2004) WebLogo: a sequence logo generator. Genome Res 14, 1188–1190. 15173120 10.1101/gr.849004 PMC419797

[70] Troshin PV , Procter JB & Barton GJ (2011) Java bioinformatics analysis web services for multiple sequence alignment – JABAWS:MSA. Bioinformatics 27, 2001–2002. 21593132 10.1093/bioinformatics/btr304 PMC3129525

